# Few-layer bifunctional metasurfaces enabling asymmetric and symmetric polarization-plane rotation at the subwavelength scale

**DOI:** 10.1038/s41598-024-62073-4

**Published:** 2024-06-13

**Authors:** Mutlu Gokkavas, T. F. Gundogdu, Ekmel Ozbay, Andriy E. Serebryannikov

**Affiliations:** 1 Nanotechnology Research Center (NANOTAM), Bilkent University, 06800 Ankara, Turkey; 2 Department of Physics, Department of Electrical Engineering, National Institute of Materials Sciences and Nanotechnology (UNAM), Bilkent University, 06800 Ankara, Turkey; 3 Division of Physics of Nanostructures, ISQI, Faculty of Physics, Adam Mickiewicz University, 61-614 Poznan, Poland

**Keywords:** Materials science, Electrical and electronic engineering

## Abstract

We introduce and numerically validate the concept of few-layer bifunctional metasurfaces comprising two arrays of quasiplanar subwavelength resonators and a middle grid (array of rectangular holes) that offer both symmetric and asymmetric transmissions connected, respectively, with symmetric and asymmetric polarization-plane rotation functionalities. The proposed structures are thinner than $\lambda /7$ and free of diffractions. Usually, the structure’s symmetry or asymmetry, i.e. unbroken or broken spatial inversion symmetries, are considered for metasurfaces as prerequisites of the capability of symmetric or asymmetric conversion of linearly polarized waves, respectively. Due to the achieved adjustment of the resonances enabling the rotation of the polarization plane simultaneously for both orthogonal polarizations of the incident wave, the symmetric polarization-plane rotation functionality can be obtained within one subwavelength band, whereas the asymmetric polarization-plane rotation functionality associated with the asymmetric transmission is obtained within another subwavelength band. This combination of the functionalities in one subdiffraction structure is possible due to the optimal choice of the grid parameters, since they may strongly affect the coupling between the two resonator arrays. Although normal incidence is required for the targeted bifunctionality, the variations of the incidence angle can also be exploited for the enrichment of the overall functional capability. Variations of the polarization angle give another important degree of freedom. The connection between the polarization-angle dependence of cross-polarized transmission and capability of symmetric and asymmetric polarization-plane rotation functionalities is highlighted. The feasible designs of the bifunctional metasurfaces are discussed.

## Introduction

Half-wave plate (HWP) functionality, i.e. a $90^{\circ }$ rotation of the polarization plane for a linearly polarized (LP) incident wave in transmission mode belongs to the basic scenarios of electromagnetic waves manipulation. The traditional approach to polarization-plane rotation (PPR) is to use a slab of a homogeneous anisotropic material. In the 2000s quasiplanar metamaterials have been suggested for the microwave frequency range. They perform various types of polarization manipulation due to the specific geometrical shape and location of the structural components that may enable bianisotropy/chirality at the subwavelength scale^1–5^. This means that the thickness of the slab of metamaterial comprising the properly designed subwavelength meta-atoms is (much) less than the incident-wave wavelength; at the same time, the metastructure can be free of diffractions at least within the selected range of incidence angles. In recent years, when metasurfaces have been raising from the area of metamaterials, the quasiplanar metamaterials enabling polarization manipulation started to be called few-layer metasurfaces. The above-mentioned bianisotropic/chiral metamaterials may emulate HWP functionality, although the underlying mechanism of PPR differs from that in the classical HWP. That is why the new terminology like metasurface (based) HWP, or similar, is widely used^6–8^. In addition to the designs based on subwavelength resonators, resonator-free, e.g. grid-based few-layer asymmetric metastructures, which are highly capable of polarization manipulation, have been proposed^9–12^. Moreover, designs comprising both grids and resonators (or phase shift enabling resonant components) have been explored^12–14^.

Along with PPR, diodelike asymmetric transmission (AT) is simultaneously achieved for LP waves in such Lorentz-reciprocal metastructures, because their symmetry breaking (with respect to the midplane in the propagation direction) is the necessary condition for obtaining the rotation of the polarization plane at normal incidence, which is simultaneously a condition for AT^1,2,11,14–24^. As shown earlier, breaking the symmetry may also lead to AT in such reciprocal structures that are not capable of polarization manipulation, as may occur, for instance, due to the transmission/reflection channels created by higher diffraction orders^25,26^ and/or the effects of spatially shaped beams^2,27^. It is noteworthy that polarization conversion is probably the only subdiffraction mechanism of AT in free-standing structures. Even single-layer unitary (i.e. nongradient) metasurfaces may yield conversion of an LP wave to a circularly polarized (CP) one^28,29^ and diverse scenarios of polarization manipulation, in reflection mode^29–31^. Moreover, AT can be obtained by means of manipulation by CP waves in a single-layer unitary chiral metastructure, although a high forward-to-backward transmission contrast is not (easily) achievable^32–34^. In addition, it is worth mentioning the geometric-phase (gradient) metasurfaces, which enable a myriad of scenarios of polarization manipulation that involve both LP and CP waves^35–40^.

The interest in *multifunctionality* has been growing in recent years, since it offers unprecedented opportunities for unification and integration^27,29,41–48^. According to this concept, two functionalities can be achieved in the different (often - closely spaced) frequency ranges or incidence-angle ranges, or even merged into one at a given angle and frequency^49^. Even a richer variety of the scenarios can be offered for multifunctional operation by the structures comprising the actively tunable materials^10,50–55^. In this case, two or more functionalities can be obtained at the same frequency and angle of incidence, for different values of the biasing parameter or different intensities of pumping.

In the present paper, we introduce and numerically validate the bifunctional metasurfaces comprising the U-shaped subwavelength resonators and the middle grid (rectangular-hole array) that may enable both the symmetric and asymmetric PPR functionalities in one microwave structure at the subwavelength scale; correspondingly, symmetric transmission will be targeted for the former but asymmetric transmission for the latter, while the same LP illumination is used for the two opposite incidence directions. As the conceptual pre-prototype, the few-layer metastructures comprising two arrays of metallic subwavelength resonators (one of which is rotated with respect to the other) and a metallic grid are selected, which may enable diodelike AT due to the joint effect of chirality and tunneling^18,56^, but they have not yet been shown to yield the symmetric PPR functionality. Owing to the new degree of freedom that is introduced by the grid, such metastructures are expected to be good candidates to combine various elementary functionalities and, therefore, reinforce the overall multifunctionality potential even without entirely new design solutions. The targeted bi-functionality will be achieved by adjusting the geometrical parameters of the structure’s components, and it will be shown that the achieved combination of the selected functionalities is not accidental. To compare, in our earlier studies of polarization manipulating metasurfaces with the middle grid, the effect of spectral overlap of strong cross-polarization components and relevant symmetric-asymmetric PPR functionality have been found for neither the similar metastructures with U-shaped subwavelength resonators^57^ nor the metastructures comprising complementary U-shaped resonators^56^. The present paper is organized as follows. In Sect. "Results and discussion", the basic features of few-layer nonsymmetric metasurfaces that comprise two arrays of subwavelength resonators are revisited and a route to nonsymmetric metasurfaces enabling a nearly symmetric PPR functionality in one transmission band and AT within the other bands is identified. Then, some aspects related to the use of nonzero angles of incidence, arbitrary polarization angles, circular polarization, and design feasibility will be discussed. The last section presents a short conclusion.

## Results and discussion

### Basic formulas and structure geometry

**Figure 1:**
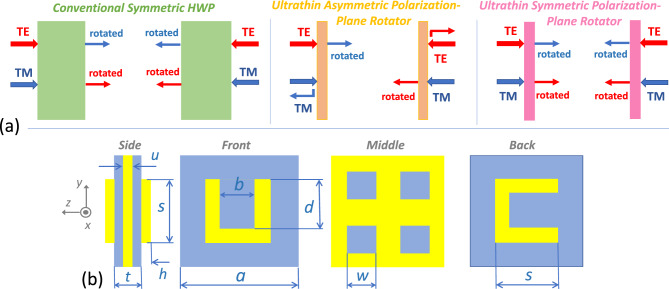
(**a**) Schematics of the operating scenarios of a conventional HWP (left plot), an ultrathin metasurface enabling asymmetric PPR (middle plot), and an ultrathin metasurface enabling symmetric PPR (right plot); (**b**) schematic of *basic geometry* of unit cell (one period) of metasurface having the following sizes: $s=10~\hbox {mm}$, $b=4~\hbox {mm}$, $d=7~\hbox {mm}$, $h=0.75~\hbox {mm}$, $a=22~\hbox {mm}$, $u=0.5~\hbox {mm}$, $t=3~\hbox {mm}$, *w* differs for different designs; the parts shown in gray-blue are made of a dielectric; the parts shown in yellow are made of copper with conductivity $\sigma =5.96\times {10^7}~\hbox {S/m}$. The spacers between each of resonator arrays and the middle grid have the same thickness $q=(t-u)/2$.

In Fig. 1a, the aforementioned operating scenarios of the classical HWP and the metasurface capable of PPR are schematically shown. Figure 1b presents details of geometry of a metasurface’s unit cell. The structure is assumed to be illuminated by an LP wave incident at the angle $\theta$, which is measured from the *z* axis in the counter-clockwise direction in the (*x*,*z*)-plane. Front-side (forward) illumination corresponds to propagation along the $-z$ direction and is indicated by $\rightarrow$. In turn, back-side (backward) illumination corresponds to the propagation along the opposite, i.e. in the $+z$ direction and it is indicated by $\leftarrow$.

In the general case, the incident and transmitted waves are connected by the following matrix equations:

1 $$\begin{aligned} \begin{pmatrix} T_x^{\rightarrow } \\ T_y^{\rightarrow } \\ \end{pmatrix} = \begin{pmatrix} \tau _{xx}^{\rightarrow } &{} \tau _{xy}^{\rightarrow } \\ \tau _{yx}^{\rightarrow } &{} \tau _{yy}^{\rightarrow } \\ \end{pmatrix} \begin{pmatrix} I_x \\ I_y \\ \end{pmatrix}, \hspace{15 mm} \begin{pmatrix} T_x^{\leftarrow } \\ T_y^{\leftarrow } \\ \end{pmatrix} = \begin{pmatrix} \tau _{xx}^{\leftarrow } &{} \tau _{xy}^{\leftarrow } \\ \tau _{yx}^{\leftarrow } &{} \tau _{yy}^{\leftarrow } \\ \end{pmatrix} \begin{pmatrix} I_x \\ I_y \\ \end{pmatrix}, \end{aligned}$$

where $I_x$ and $I_y$ are the complex amplitudes of the *x* and *y* polarized incident waves, respectively, and $T_x$ and $T_y$ are the complex amplitudes of the *x* and *y* polarized transmitted waves, respectively. The components of *T* matrix describe the contribution of the incident wave to the co-polarized ($\tau _{xx}^{\rightarrow }$,$\tau _{yy}^{\rightarrow }$,$\tau _{xx}^{\leftarrow }$, $\tau _{yy}^{\leftarrow }$) and cross-polarized ($\tau _{xy}^{\rightarrow }$,$\tau _{yx}^{\rightarrow }$,$\tau _{xy}^{\leftarrow }$,$\tau _{yx}^{\leftarrow }$) transmission components. Owing to the Lorentz reciprocity^20,58^, for the co-polarized transmission, we have $\vert \tau _{yy}^\rightarrow \vert =\vert \tau _{yy}^\leftarrow \vert$, $\vert \tau _{xx}^\rightarrow \vert =\vert \tau _{xx}^\leftarrow \vert$, and so we further omit the arrows indicating direction. Moreover, $\vert \tau _{yy}\vert =\vert \tau _{xx}\vert$ due to the specific symmetry of the studied structure. For the cross-polarized transmission, Lorentz reciprocity and symmetry properties result in $\vert \tau _{xy}^\rightarrow \vert =\vert \tau _{yx}^\leftarrow \vert$ and $\vert \tau _{yx}^\rightarrow \vert =\vert \tau _{xy}^\leftarrow \vert$. More information about the effects exerted by the structure’s symmetry and its breaking can be found in Ref. ^20^. To quantify the extent of asymmetry in transmission, *asymmetric transmission contrast* can be introduced as follows:

2 $$\begin{aligned} ATC=\hbox {max}(\eta _i/\zeta _i,\zeta _i/\eta _i), \end{aligned}$$

where $i=x,y$, i.e. we take $\eta _x=\vert \tau _{yx}^{\rightarrow }\vert ^2+\vert \tau _{xx}\vert ^2$ and $\zeta _x=\vert \tau _{yx}^{\leftarrow }\vert ^2+\vert \tau _{xx}\vert ^2$ for $i=x$, and $\eta _y=\vert \tau _{xy}^{\rightarrow }\vert ^2+\vert \tau _{yy}\vert ^2$ and $\zeta _y=\vert \tau _{xy}^{\leftarrow }\vert ^2+\vert \tau _{yy}\vert ^2$ for $i=y$, i.e. for the *x* and *y* polarized incident waves, respectively. In turn, the polarization conversion ratio is introduced in forward illumination case as

3 $$\begin{aligned} PCR_x^{\rightarrow }=\vert \tau _{yx}^{\rightarrow }\vert ^2/\eta _x,\hspace{10 mm} PCR_y^{\rightarrow }=\vert \tau _{xy}^{\rightarrow }\vert ^2/\eta _y. \end{aligned}$$

In the backward illumination case, $PCR_x^{\leftarrow }$ and $PCR_y^{\leftarrow }$ are obtained, respectively, from $PCR_x^{\rightarrow }$ and $PCR_y^{\rightarrow }$ by substituting $\eta _x$ by $\zeta _x$ and $\eta _y$ by $\zeta _y$, and $\rightarrow$ by $\leftarrow$ in the numerator, so that $PCR_y^{\leftarrow }=PCR_x^{\rightarrow }$ and $PCR_x^{\leftarrow }=PCR_y^{\rightarrow }$.

In particular, if a strongly pronounced AT is targeted, we need in the ideal case $ATC=\infty$ and either $PCR_y^{\rightarrow }\approx {1}$ (but $PCR_x^{\rightarrow }\approx {0}$) when $\vert \tau _{xy}^{\rightarrow }\vert \gg \vert \tau _{xy}^{\leftarrow }\vert$ and $\vert \tau _{xy}^{\rightarrow }\vert \gg \vert \tau _{yy}\vert$, or $PCR_x^{\rightarrow }\approx {1}$ (but $PCR_y^{\rightarrow }\approx {0}$) when $\vert \tau _{xy}^{\rightarrow }\vert \ll \vert \tau _{xy}^{\leftarrow }\vert$ and $\vert \tau _{xx}\vert \ll \vert \tau _{xy}^{\leftarrow }\vert$. In turn, for the symmetric PPR, we have to weaken the co-polarized components and strengthen the cross-polarized components, but then we need $\vert \tau _{xy}^{\rightarrow }\vert \approx \vert \tau _{xy}^{\leftarrow }\vert$ and $\vert \tau _{yx}^{\rightarrow }\vert \approx \vert \tau _{yx}^{\leftarrow }\vert$ that leads, in such a nearly ideal case, to $ATC\approx {1}$ and $PCR_x^{\rightarrow }\approx {PCR_y^{\rightarrow }\approx {1}}$. Therefore, the difference between the symmetric and asymmetric PPR functionalities can be quantified in terms of *ATC* and *PCR*.

### Revisiting the transmission features of metasurfaces with and without middle grids

First, let us revisit the general properties of few-layer metasurfaces that are best known as enablers of AT for LP waves, which appears in the direct connection with the asymmetric PPR functionality. Their necessary components include two arrays of split-ring resonators, or subwavelength resonators of a similar shape, which are separated by a thin spacer made of a dielectric with permittivity $\varepsilon _s$. The back-side array represents a $90^{\circ }$ rotated version of the front-side array; see Fig. 1b.

**Figure 2:**
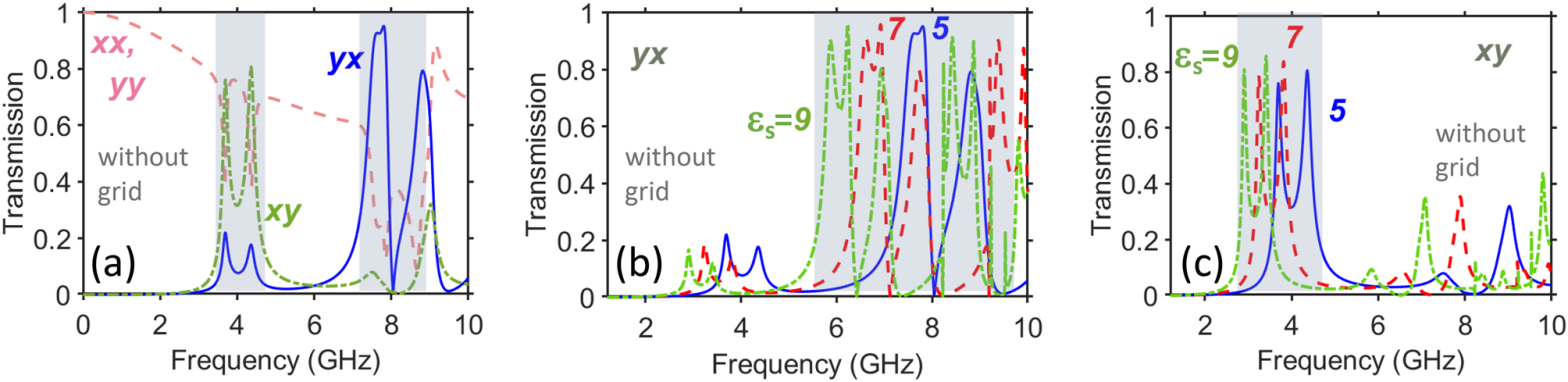
(**a**) Demonstration of basic transmission features of metasurfaces without a middle grid: spectrums of $\vert \tau _{xx}\vert =\vert \tau _{yy}\vert$ (rose dashed line), $\vert \tau _{xy}^{\rightarrow }\vert =\vert \tau _{yx}^{\leftarrow }\vert$ (green dash-dotted line), $\vert \tau _{yx}^{\rightarrow }\vert =\vert \tau _{xy}^{\leftarrow }\vert$ (blue solid line) at $\varepsilon _s=5$. Effect of $\varepsilon _s$ on cross-polarized transmission components whose (in)equality is crucial for resulting PPR functionality: (**b**) $\vert \tau _{yx}^{\rightarrow }\vert =\vert \tau _{xy}^{\leftarrow }\vert$ at $\varepsilon _s=5$ (blue solid line), $\varepsilon _s=7$ (red dashed line), and $\varepsilon _s=9$ (green dash-dotted line); (**c**) same as (**b**) but for $\vert \tau _{xy}^{\rightarrow }\vert =\vert \tau _{yx}^{\leftarrow }\vert$. The grid is assumed to be replaced, compared with Fig. 1b, by a homogeneous layer with permittivity $\varepsilon _s$, i.e. a single spacer between two arrays of resonators has thickness *t*; $\theta =0^{\circ }$. Semi-transparent gray rectangles indicate the *f*-ranges, in which strong cross-polarized components and related AT may appear. Indicators *xy*, *yx*, *xx*, *yy* correspond to forward illumination.

Figure 2 presents the spectrums of the co-polarized transmission component, $\vert \tau _{xx}\vert =\vert \tau _{yy}\vert$, and the cross-polarized components, $\vert \tau _{xy}^{\rightarrow }\vert =\vert \tau _{yx}^{\leftarrow }\vert$ and $\vert \tau _{yx}^{\rightarrow }\vert =\vert \tau _{xy}^{\leftarrow }\vert$, for the grid-free structures with the geometrical parameters introduced in Fig. 1b. The lowest-frequency subwavelength resonances yield the cross-polarized transmission band around $f=4$ GHz [see Fig. 2a,c]. In this case, the magnetic component of the incident wave that is parallel to the apertures of the front-side resonators is responsible for strong polarization conversion [see $\vert \tau _{xy}^{\rightarrow }\vert$ in Fig. 2c]. At the same time, when an electric component is parallel to the apertures, weak cross-polarized effects are obtained near 4 GHz [see $\vert \tau _{yx}^{\rightarrow }\vert$ in Fig. 2b]. Note that the spectral positions of the resonances enabling cross-polarized transmission nearly coincide with those for co-polarized transmission in the case of two coupled parallel (i.e., not rotated) arrays but with the same orientation of the resonators as that of the front-side resonators in the studied metasurface; see Figs. 1–3 in Ref.^59^ for comparison.

The situation is different for the second band located at $f=7.5$ GHz. In this case, strong cross-polarization effects are observed for the *x*-polarized incident waves, i.e. when the electric component of the incident wave is parallel to the apertures of the front-side resonators. Now, we obtain $ATC\approx 37$ and $PCR_x^{\rightarrow }=0.974$ at $f=7.75$ GHz. To compare, in the first band, $ATC\approx 4.3$ and $PCR_y^{\rightarrow }=0.81$ at $f=4.28$ GHz. An important feature observed in Fig. 2b,c is that the spectral ranges of strong cross-polarized transmission are approximately *scalable* by varying $\varepsilon _s$, similarly to Refs.^57,60^. It is worth noting that passbands comprising two closely spaced maximums are typical for the metastructures containing two coupled arrays of subwavelength resonators that are placed at the front and back sides of the structure and belong to the same unit cell (period); a coupling of this kind can be explained by using the Lagrange model^61^.

**Figure 3:**
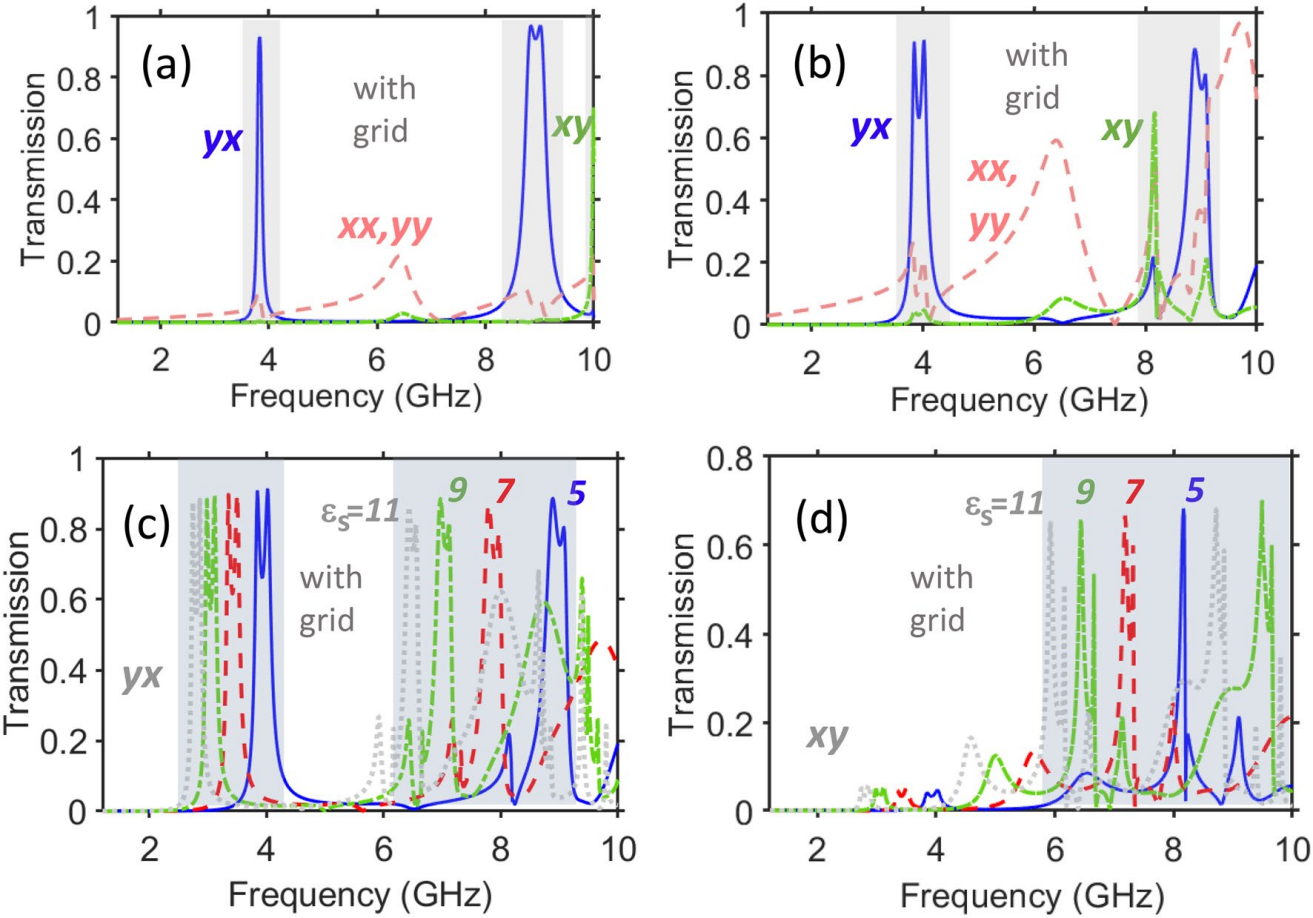
Demonstration of basic transmission features of metasurfaces with a middle grid for two values of *w*: (**a**) spectrums of $\vert \tau _{xx}\vert =\vert \tau _{yy}\vert$ (rose dashed line), $\vert \tau _{xy}^{\rightarrow }\vert =\vert \tau _{yx}^{\leftarrow }\vert$ (light-green dash-dotted line), and $\vert \tau _{yx}^{\rightarrow }\vert =\vert \tau _{xy}^{\leftarrow }\vert$ (blue solid line), at $\varepsilon _s=5$ for a grid-containing structure with $w=5$ mm [see Fig. 1b]; (**b**) same as (**a**) but at $w=7$ mm, and effect of $\varepsilon _s$ on cross-polarized transmission components whose (in)equality is crucial for resulting PPR functionality: (**c**) $\vert \tau _{yx}^{\rightarrow }\vert =\vert \tau _{xy}^{\leftarrow }\vert$ at $\varepsilon _s=5$ (blue solid line), $\varepsilon _s=7$ (red dashed line), $\varepsilon _s=9$ (light-green dash-dotted line), and $\varepsilon _s=11$ (gray dotted line), at $w=7$ mm; (**d**) same as (**c**) but for $\vert \tau _{xy}^{\rightarrow }\vert =\vert \tau _{yx}^{\leftarrow }\vert$; $\theta =0^{\circ }$. Semi-transparent gray rectangles indicate the *f*-ranges in which strong cross-polarized components and related AT may appear. Indicators *xy*, *yx*, *xx*, *yy* correspond to forward illumination.

From the asymmetric PPR and, therefore, from the AT perspective, the disadvantages of this design and, often, other two-array metastructures is that the unwanted co-polarized transmission component can not be well suppressed, as well as the second cross-polarized component, which is also unwanted for AT, so that *ATC* is lower than may be required when highly-efficient AT is targeted. It is noteworthy that optimization in terms of the material and geometric parameters is possible so that it may yield higher *PCR* and *ATR*; compare to Ref.^57^. From the symmetric-PPR perspective, the resonances excited by each of the *x* and *y* polarized electromagnetic waves typically have different spectral locations within the subwavelength range, so that they are not suitable for a symmetric functionality (i.e. the one being identical for the front-side and back-side illuminations at given *f*).

A useful modification of the classical two-array based polarization-rotating structure like the one in Fig. 1b, has been proposed in the 2010s by introducing a metallic grid between the two resonator arrays that may allow for a partial mitigation of the aforementioned disadvantages^56,57^. Adding a grid yields an additional degree of freedom for controlling a coupling between the arrays. The resulting mechanism is typically based in this case on the combination of chirality and tunneling^18^. As observed in Fig. 3a,b for the basic geometry in case of the *x*-polarized incident waves, two cross-polarized transmission bands with high $\vert \tau _{yx}^{\rightarrow }\vert$ appear at $f=4$ GHz and $f=9$ GHz, i.e. the incident electric field is parallel to the apertures of front-side resonators. This behavior differs from that for the grid-free metastructure in Fig. 2, the corresponding single array, and examples presented for the grid-free (mesh-free) case in Ref.^57^. It occurs due to the effects exerted by the metallic grid. At the same time, the second cross-polarized component, i.e. $\tau _{xy}^{\rightarrow }$ can also show quite high efficiency, as observed in Fig. 3a at $f=10$ GHz, and in Fig. 3b near $f=8$ GHz. These resonances are excited when the magnetic field component of the incident wave is parallel to the apertures of front-side resonators. The observed high sensitivity to the variations in *w* may indicate a strong local field enhancement in the holes and near the grid.

Interestingly, the transmission maximum for the *y*-polarized incident waves is strongly sensitive to the variations in *w*, i.e. it is shifted from 10 GHz down to 8 GHz, while both cross-polarized transmission bands for the *x*-polarized incident waves keep their spectral locations nearly the same. Moreover, it is observed that the maximum can be located at both smaller and larger values of *f* compared to the band of high $\vert \tau _{yx}^{\rightarrow }\vert$. This indicates that the overlap of resonances for the two orthogonal polarization states of the incident wave can be achieved by means of adjusting the *w*-value that is expected to yield a nearly symmetric PPR functionality at the subwavelength scale. Therefore, we shall search for the resonances which coincide despite the fact that the broken spatial-inversion symmetry leads, in the general case, to the illumination-direction dependent spectral positions of the polarization-conversion resonances. In other words, we need a scenario in which AT enabled by the asymmetric-PPR functionality and symmetric transmission enabled by the symmetric-PPR functionality co-exist in different parts of the subwavelength range in contrast with the most typical scenario in which two or even more AT bands are only occurring within this range. It is noteworthy that the dramatic changes in the transmission scenario, compared to the grid-free metastructure, can be explained in terms of equivalent impedances and/or effective material parameters (that is, however, beyond the current scope). Figure 3c,d shows, similarly to Fig. 2b,c, that the resonance frequencies are scalable for the cross-polarized components by means of variations in $\varepsilon _s$. Indeed, the basic features are retained, while corresponding frequencies are changed approximately as $f\propto {1}/\sqrt{\varepsilon _s}$.

### Designing the structures with asymmetric and symmetric functionalities

Based on the results presented in Fig. 3, it may be expected that the maxima of $\vert \tau _{xy}^{\rightarrow }\vert$ and $\vert \tau _{yx}^{\rightarrow }\vert$ can have the same spectral positions, as desired for nearly the same (i.e. symmetric) rotation of the polarization plane for both forward and backward illumination directions. It has been realized that such an adjustment can be achieved by means of variations in the middle grid sizes. Clearly, this possibility is absent in the polarization-converting metastructures without a middle grid.

**Figure 4:**
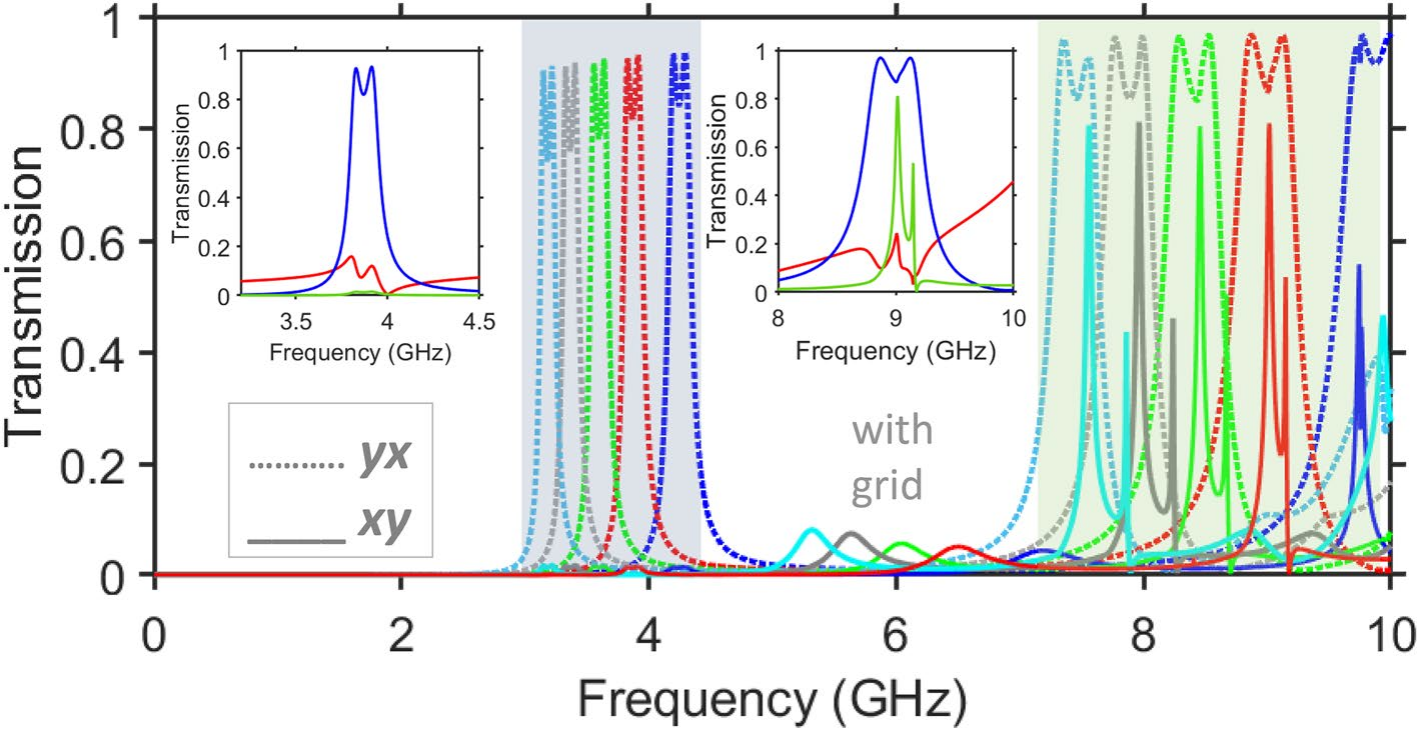
Demonstration of the coexistence of nearly equal and inequal cross-polarized components in one metastructure, at different values of $\varepsilon _s$: spectrums of $\vert \tau _{xy}^{\rightarrow }\vert =\vert \tau _{yx}^{\leftarrow }\vert$ (solid lines) and $\vert \tau _{yx}^{\rightarrow }\vert =\vert \tau _{xy}^{\leftarrow }\vert$ (dashed lines) at $\varepsilon _s=4$ (blue lines), $\varepsilon _s=5$ (red lines), $\varepsilon _s=6$ (light-green lines), $\varepsilon _s=7$ (gray lines), $\varepsilon _s=8$ (light-blue lines) for the grid-containing structure with $w=6$ mm and the same remaining parameters as in Fig. 3. Left and right insets are fragments of the spectrums of $\vert \tau _{xx}\vert =\vert \tau _{yy}\vert$ (red lines), $\vert \tau _{yx}^{\rightarrow }\vert =\vert \tau _{xy}^{\leftarrow }\vert$ (blue lines), and $\vert \tau _{xy}^{\rightarrow }\vert =\vert \tau _{yx}^{\leftarrow }\vert$ (green lines), in the vicinity of $f=4$ GHz and $f=9$ GHz, respectively, for $\varepsilon _s=5$; $\theta =0^{\circ }$. Semi-transparent gray and green rectangles indicate, respectively, the *f*-ranges in which either only asymmetric or both symmetric and asymmetric PPR are possible.

Figure 4 presents the magnitude of the cross-polarized transmission components, $\vert \tau _{xy}^{\rightarrow }\vert$ and $\vert \tau _{yx}^{\rightarrow }\vert$ vs. *f* in the case when both quantities can exceed 0.75. It is obtained here by taking $w=6$ mm. You can see that, in a wide $\varepsilon _s$ range, the narrow peak of high $\vert \tau _{xy}^{\rightarrow }\vert$ is located within a wide band of high $\vert \tau _{yx}^{\rightarrow }\vert$.

Depending on the choice of $\varepsilon _s$, $\hbox {max}\vert \tau _{xy}^{\rightarrow }\vert$ can coincide with the second maximum of $\vert \tau _{yx}^{\rightarrow }\vert$ in the second band, like it occurs between 7 and 8 GHz at $\varepsilon _s=8$, or be located between the two maximums of $\vert \tau _{yx}^{\rightarrow }\vert$ in the second band, as happens at $\varepsilon _s=5$. Notably, the coincidence of spectral positions of the maximums can not be achieved starting nearly from $\varepsilon _s=10$. On the other hand, $\hbox {max}\vert \tau _{xy}^{\rightarrow }\vert <0.7$ at $\varepsilon _s<5$ for the chosen sizes. From the obtained results, it follows that the case of $\varepsilon _s=5$ is most suitable. However, the second-stage optimization is required for the rigorous equality of $\vert \tau _{xy}^{\rightarrow }\vert$ and $\vert \tau _{yx}^{\rightarrow }\vert$, along with their maximization, it will be considered in Sect. “Toward design adjustment”. At the same time, the usual scenario of AT, which is yielded by the asymmetric PPR regime, is obtained in the first band, i.e. in the vicinity of $f=4$ GHz.

The details of both symmetric and asymmetric PPR bands are shown in the insets in Fig. 4. Indeed, a moderately efficient AT is achieved in the first band, where $ACT\approx 71$ at $f=3.83$ GHz, whereas $PCR_y^{\rightarrow }=0.986$; see the left inset in Fig. 4. Strictly speaking, AT can appear at $\varepsilon _s=5$ not only within the first polarization-conversion band but also within the second band, i.e. near $f=8.9$ GHz. In other words, asymmetric and symmetric PPR functionalities can be obtained in the closely spaced spectral regimes, i.e. in the vicinity of $f=8.9$ GHz. In terms of *ATC* and *PCR*, the difference between these functionalities is well seen: $ATC\approx 58$ and $PCR_y^{\rightarrow }=0.99$ at the first maximum of $\vert \tau _{yx}^{\rightarrow }\vert$ in the second band, and $ATC=1.12$ and $PCR_y^{\rightarrow }=0.997$ between the first and the second maximums of $\vert \tau _{yx}^{\rightarrow }\vert$ in the same band. The obtained results show that the main goal is achievable: the symmetric and asymmetric PPR functionalities can be obtained at the subwavelength scale in one metastructure. Some examples of the field distribution are presented in Supplementary Material^62^.

### Exploring angular effects

**Figure 5:**
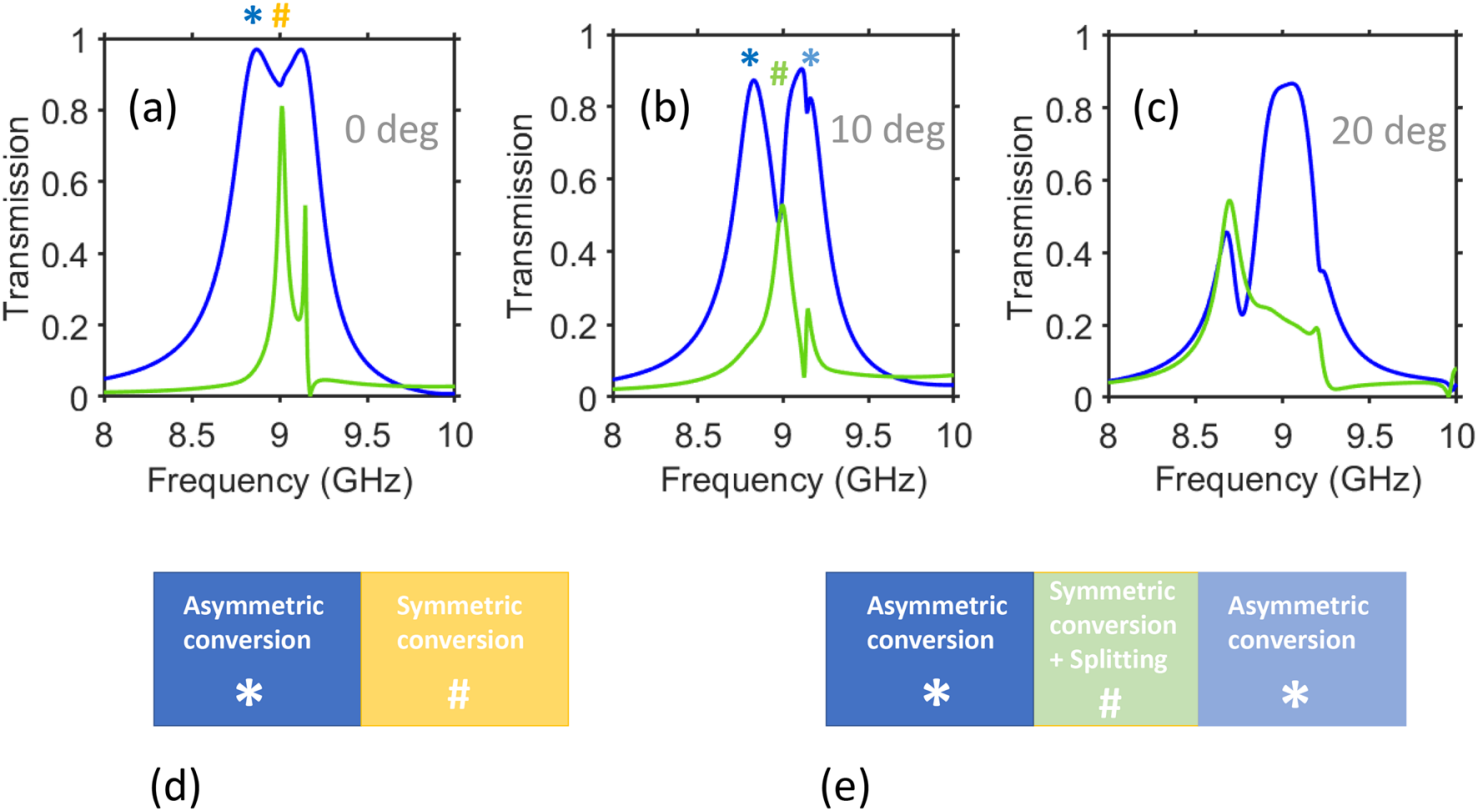
Effects of $\theta$ variation for the structure from Fig. 4: spectrums of $\vert \tau _{yx}^{\rightarrow }\vert =\vert \tau _{xy}^{\leftarrow }\vert$ (blue lines) and $\vert \tau _{xy}^{\rightarrow }\vert =\vert \tau _{yx}^{\leftarrow }\vert$ (green lines) at (**a**) $\theta =0^{\circ }$, (**b**) $\theta =10^{\circ }$, (**c**) $\theta =20^{\circ }$, for $\varepsilon _s=5$; the nearly symmetric and asymmetric PPR regimes are indicated by hashtags and asterisks, respectively. Schematics showing spectral location of the above-mentioned PPR regimes with respect to each other for (**d**) $\theta =0^{\circ }$ and (**e**) $\theta =10^{\circ }$.

This section is dedicated to the effects arising due to the variations in $\theta$ and in polarization angle, $\phi$ [it is measured from *x* axis in (*x*,*y*)-plane]. As follows from the obtained results (both shown and not shown), there may be diverse scenarios of changing the *f*-dependencies of $\vert \tau _{yx}^{\rightarrow }\vert$ and $\vert \tau _{xy}^{\rightarrow }\vert$ while varying $\theta$. Demonstration of all of them is not a purpose; the emphasis is put here on the selected scenarios. In Fig. 5, the results are presented for the same structure as in Fig. 4, at three values of $\theta$. Consideration is restricted to the second PPR band, within which a nearly symmetric functionality can be obtained. At $\theta =10^{\circ }$, the two maximums of $\vert \tau _{yx}^{\rightarrow }\vert$ are still strong but more strongly separated from each other than at $\theta =0^{\circ }$; $\vert \tau _{yx}^{\rightarrow }\vert$ and $\vert \tau _{xy}^{\rightarrow }\vert$ are now less than 0.6 in the vicinity of $f\approx {9}$ GHz, so that the resulting functionality can be considered rather as merging of PPR and splitter functionalities. Two AT regimes with $\hbox {max}\vert \tau _{yx}^{\rightarrow }\vert >0.9$ occur at $\theta =10^{\circ }$ in the neighboring *f*-ranges, i.e. around 8.8 GHz and 9.1 GHz. At $\theta =20^{\circ }$, a nearly symmetric but low-efficiency PPR is obtained at $f\approx {8.6}$ GHz, while the asymmetric PPR functionality enabling a low-contrast AT is achieved here around $f=9$ GHz. Therefore, not only the choice of the grid sizes but also that of the angle of incidence is crucial for obtaining of high-efficiency symmetric and asymmetric PPR functionalities in one metastructure.

**Figure 6:**
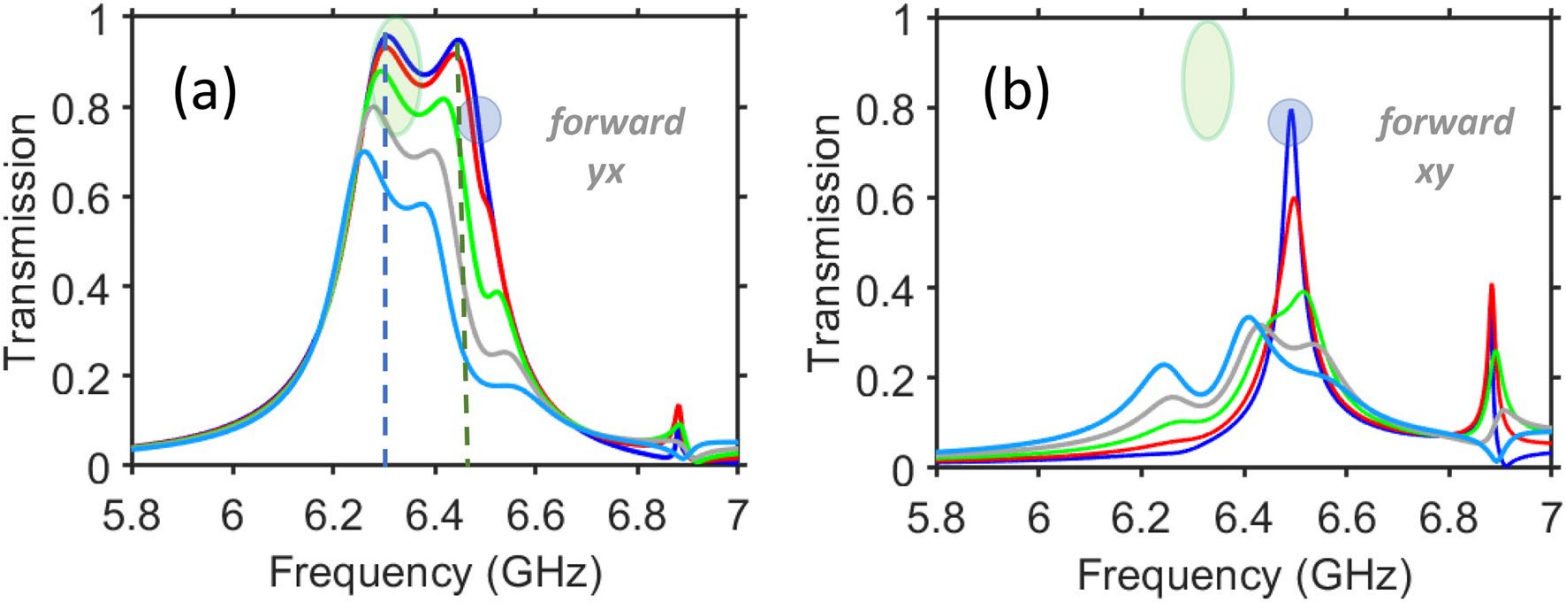
Effects of $\theta$ on cross-polarized transmission at higher permittivity of the spacers: spectrums of (**a**) $\vert \tau _{yx}^{\rightarrow }\vert =\vert \tau _{xy}^{\leftarrow }\vert$ and (**b**) $\vert \tau _{xy}^{\rightarrow }\vert =\vert \tau _{yx}^{\leftarrow }\vert$ for $\varepsilon _s=11.4$ and the same remaining parameters as in Fig. 4, at $\theta =0^{\circ }$ (blue lines), $\theta =10^{\circ }$ (red lines), $\theta =20^{\circ }$ (light-green lines), $\theta =30^{\circ }$ (gray lines), $\theta =40^{\circ }$ (light-blue lines). Vertical dashed lines indicate two spectral regimes that are suitable for low-pass spatial filtering. Greenish ellipses and bluish circles indicate high-contrast AT regime and symmetric transmission regime with $\vert \tau _{yx}^{\rightarrow }\vert =\vert \tau _{xy}^{\rightarrow }\vert$, respectively.

To further explore the effects of $\theta$ variation, Fig. 6 presents $\vert \tau _{yx}^{\rightarrow }\vert$ and $\vert \tau _{xy}^{\rightarrow }\vert$ vs. *f* for the second PPR band of the metastructure that differs from the one in Fig. 5 only in the spacers’ permittivity, which is taken here as $\varepsilon _s=11.4$. The way of results presentation also differs from that in Fig. 5, that is needed for better visibility of the basic features which just partially coincide with those in Fig. 5. As observed for the dependencies of $\vert \tau _{yx}^{\rightarrow }\vert$ vs. *f*, the increase of $\theta$ leads to the decrease of the values of $\hbox {max}\vert \tau _{yx}^{\rightarrow }\vert$ for both first and second maximums. However, they show different sensitivity to the $\theta$ variation.

In particular, $\vert \tau _{yx}^{\rightarrow }\vert =0.95$ at $\theta =0^{\circ }$, 0.65 at $\theta =40^{\circ }$, and 0.25 at $\theta =70^{\circ }$ for $f\approx 6.3$ GHz, whereas $\vert \tau _{xy}^{\rightarrow }\vert =0.95$ at $\theta =0^{\circ }$, 0.29 at $\theta =40^{\circ }$, and 0.1 at $\theta =70^{\circ }$ for $f\approx 6.45$ GHz, compare the left and right vertical dashed lines in Fig. 6a. Although both cases shown by dashed lines are appropriate for low-pass $\theta$-domain filtering, they strongly differ in terms of the $\theta$-domain bandwidth; also compare to Figs. 3 and 5 in Ref.^63^. Notably, low-pass spatial filtering [shown in Fig. 6a by vertical dashed lines] can coexist with a well-pronounced AT [shown in Fig. 6a,b by the greenish ellipses], and with the transmission of comparable (but generally inequal) intensities at forward and backward illumination. In contrast with the conventional regimes of spatial (angular) filtering, it is achieved here for the cross-polarized component, and so it can be considered as *merging* the spatial filtering and PPR functionalities in one. Details of this regime will be the subject of one of our future studies. Taking $f=6.49$ GHz, i.e. a slightly higher value than the one at which the second maximum of $\vert \tau _{yx}^{\rightarrow }\vert$ appears at $\theta =0^{\circ }$, we can obtain $\vert \tau _{yx}^{\rightarrow }\vert =\vert \tau _{xy}^{\rightarrow }\vert =0.76$, i.e. the exactly symmetric PPR functionality at the price of lowering efficiency of the cross-polarized transmission [shown in Fig. 6a,b by the bluish circles]. High *ATC* is achieved at the first maximum of $\vert \tau _{yx}^{\rightarrow }\vert$ in the considered (i.e., second) band, at least when $\theta \le 20^{\circ }$. For instance, at $f=6.3$ GHz and $\theta =0^{\circ }$, we obtain $ATC\approx 68$ and $PCR_y^{\rightarrow }=0.988$. It should also be noted that AT occurs also in the first band within a wide range of $\theta$ variation. As an example, at $f=2.75$ GHz and $\theta =0^{\circ }$, we obtain $ATC\approx 48$ and $PCR_y^{\rightarrow }=0.98$.

**Figure 7:**
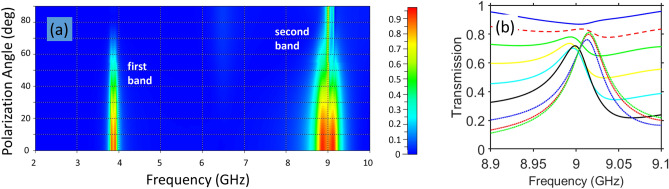
Effect of polarization angle ($\phi$) variations in connection with symmetric and asymmetric PPR functionalities: (**a**) cross-polarized transmission shown on (*f*,$\phi$)-plane for the metasurface with the same geometrical parameters as in Fig. 4; $\phi$ is varied from $0^{\circ }$ to $90^{\circ }$; $\phi =0^{\circ }$ corresponds to $\vert \tau _{yx}^{\rightarrow }\vert =\vert \tau _{xy}^{\leftarrow }\vert$, $\phi =90^{\circ }$ corresponds to $\vert \tau _{xy}^{\rightarrow }\vert =\vert \tau _{yx}^{\leftarrow }\vert$; (**b**) spectrum of the cross-polarized transmission in the vicinity of $f=9$ GHz: blue solid line - $\phi =0^{\circ }$, red dashed line - $\phi =20^{\circ }$, green solid line - $\phi =30^{\circ }$, yellow solid line - $\phi =40^{\circ }$, cyan solid line - $\phi =50^{\circ }$, black solid line - $\phi =60^{\circ }$, blue dotted line - $\phi =70^{\circ }$, red dotted line - $\phi =80^{\circ }$, green dotted line - $\phi =90^{\circ }$ (the lines are located from the top to the bottom at *f*<8.97 GHz).

An angular selectivity of another kind has been explored at variations of $\phi$ from $0^{\circ }$ to $90^{\circ }$. This variation corresponds to the gradual transition from the *x*-polarized to the *y*-polarized incident waves at the forward illumination. An example is presented in Fig. 7a. For the first band (near 4 GHz), a monotonic decrease of transmission is obtained when $\phi$ is varied from $0^{\circ }$ to $90^{\circ }$ that corresponds to the well pronounced asymmetry in transmission. For the second band (near 9 GHz), strong variations in transmission can appear within the *f*-subranges of AT, i.e. at the first maximum of $\vert \tau _{yx}^{\rightarrow }\vert$ which is observed in the right inset in Fig. 4 and in Fig. 5a, whereas the effect of variations in $\phi$ is much weaker between the maximums of the second band, as shown in Fig. 7b. This allows us to retain a relatively high capability of PPR when $\phi$ is varied over a wide range. Although $\vert \tau _{xy}^{\rightarrow }\vert$ (which corresponds to $\phi =90^{\circ }$ in Fig. 7) does not serve as the lower-transmission bound for all *f* values in the vicinity of its maximum, it can be quite close to becoming such a bound, as observed in Fig. 7b. However, the range of moderate $\phi$, e.g. the cases of $40^{\circ }$, $50^{\circ }$, $60^{\circ }$ can be said to be less appropriate for PPR functionality.

### Toward design adjustment

While the principal possibility of co-existence of the asymmetric and symmetric PPR functionalities in one metastructure was demonstrated in the previous sections, the question remains whether the desired bifunctionality can be achieved by using such dielectric materials for spacers that are commonly used for antennas and other microwave devices. Our first purpose in this section is to estimate (a) possibility of increase of $\vert \tau _{xy}^{\rightarrow }\vert$ and $\vert \tau _{yx}^{\rightarrow }\vert$ and (b) their possible equality. The second purpose is to show that the basic functionalities are robust to the variations of the material parameters and selected geometric parameters. In particular, for the commercial materials, a tolerance range of permittivity is often provided, rather than a single value.

**Figure 8:**
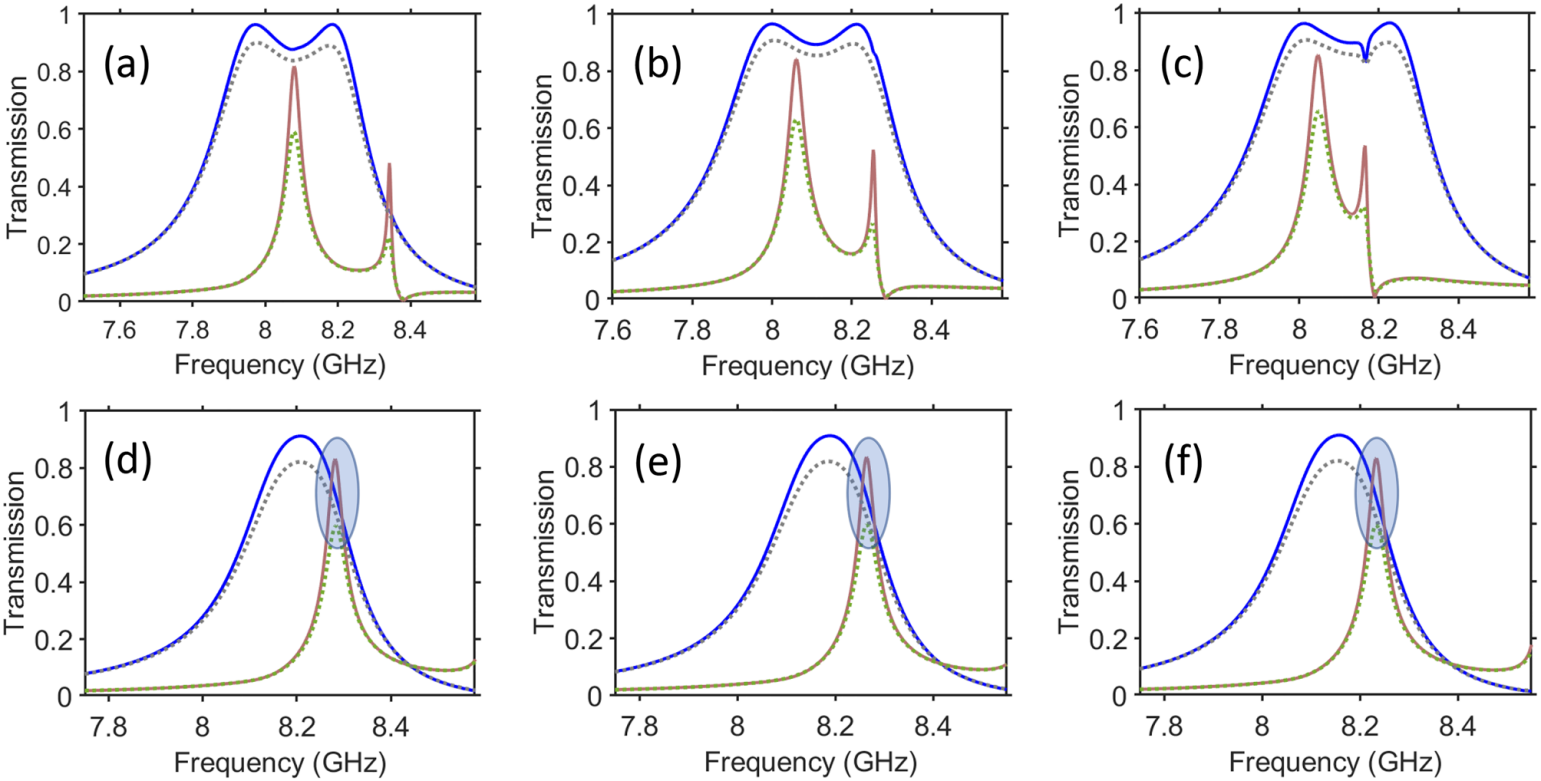
Comparison of transmission for the six selected sets of parameters: (**a**) $h=0.5~\hbox {mm}$, (**b**) $h=1.0~\hbox {mm}$, and (**c**) $h=1.5~\hbox {mm}$ when $t=3.06~\hbox {mm}$, $u=0.5~\hbox {mm}$, $w=6.1~\hbox {mm}$ and $\varepsilon _s=6.5$; (**d**) $\varepsilon _s=6.41$, (**e**) $\varepsilon _s=6.45$ and (**f**) $\varepsilon _s=6.5$ when $t=3.56~\hbox {mm}$, $u=1.0~\hbox {mm}$, $h=7\times {10^{-2}}~\hbox {mm}$, $w=6.37~\hbox {mm}$; the remaining parameters are the same as in Fig. 4; blue solid lines - $\vert \tau _{yx}^{\rightarrow }\vert =\vert \tau _{xy}^{\leftarrow }\vert$, case of lossless spacers; gray dotted lines - $\vert \tau _{yx}^{\rightarrow }\vert =\vert \tau _{xy}^{\leftarrow }\vert$, $\hbox {tan}\delta =2\times 10^{-3}$ for spacers; brown solid lines - $\vert \tau _{xy}^{\rightarrow }\vert =\vert \tau _{yx}^{\leftarrow }\vert$, case of lossless spacers; green dotted lines - $\vert \tau _{xy}^{\rightarrow }\vert =\vert \tau _{yx}^{\leftarrow }\vert$, $\hbox {tan}\delta =2\times 10^{-3}$ for spacers. Bluish ellipses indicate the cases of (nearly) equal cross-polarized transmission components.

Figure 8 presents the results for the second PPR band in the case when both spacers are made of Roger RO3006 laminate^64^. As observed in Fig. 8a–c, the change of *h* from 0.5 to 1.0 mm and then to 1.5 mm does not lead to significant changes in the spectrums of the cross-polarized components. Note that $\vert \tau _{xy}^{\rightarrow }\vert \approx {0.85}$ is observed in Fig. 8c for lossless spacers, but $\vert \tau _{yx}^{\rightarrow }\vert$ is still larger than $\vert \tau _{xy}^{\rightarrow }\vert$ at the same *f*. The scenario of (nearly) symmetric PPR, for which $\hbox {max}\vert \tau _{xy}^{\rightarrow }\vert$ is located between two maximums of $\vert \tau _{yx}^{\rightarrow }\vert$, can be obtained for various parameter sets. When losses in the spacers are taken into account ($\hbox {tan}\delta =2\times 10^{-3}$, in line with Ref.^64^), cross-polarized transmission predictably tends to weaken. As observed, $\vert \tau _{yx}^{\rightarrow }\vert$ is less sensitive to the losses than $\vert \tau _{xy}^{\rightarrow }\vert$, expectedly due to lower-Q resonances. The results presented in Fig. 6 may give us a hint as to how $\vert \tau _{xy}^{\rightarrow }\vert =\vert \tau _{yx}^{\rightarrow }\vert >0.7$ could be achieved. Indeed, the frequencies that are a bit larger than the one of $\hbox {max}\vert \tau _{yx}^{\rightarrow }\vert$ look suitable, see Fig. 8d–f. It is worth noting that the band of $\vert \tau _{yx}^{\rightarrow }\vert$ with two maximums is transformed into a single wide maximum for the chosen sets of geometric parameters, among which *h* is critical. It is demonstrated here that the regime of $\vert \tau _{xy}^{\rightarrow }\vert =\vert \tau _{yx}^{\leftarrow }\vert =\vert \tau _{yx}^{\rightarrow }\vert = \vert \tau _{xy}^{\leftarrow }\vert \approx 0.8$ (in case of lossless spacers) is robust to the variations in $\varepsilon _s$, in spite of the fact that the *f* value, at which $\hbox {max}\vert \tau _{xy}^{\rightarrow }\vert$ is obtained, is weakly changed. Although transmission is weaker if the losses are taken into account, the equality of $\vert \tau _{xy}^{\rightarrow }\vert$ and $\vert \tau _{yx}^{\rightarrow }\vert$ is still achievable at the acceptable level, e.g. 0.6. The cases of (nearly) equal cross-polarized components are shown by the bluish ellipses. Clearly, Roger RO3006 is not the only commercial material suitable for the spacers. For instance, TMM4 laminate^65^ is one more material, for which the features similar to those in Fig. 8 have been numerically demonstrated (not shown).

### Involving circular polarization

**Figure 9:**
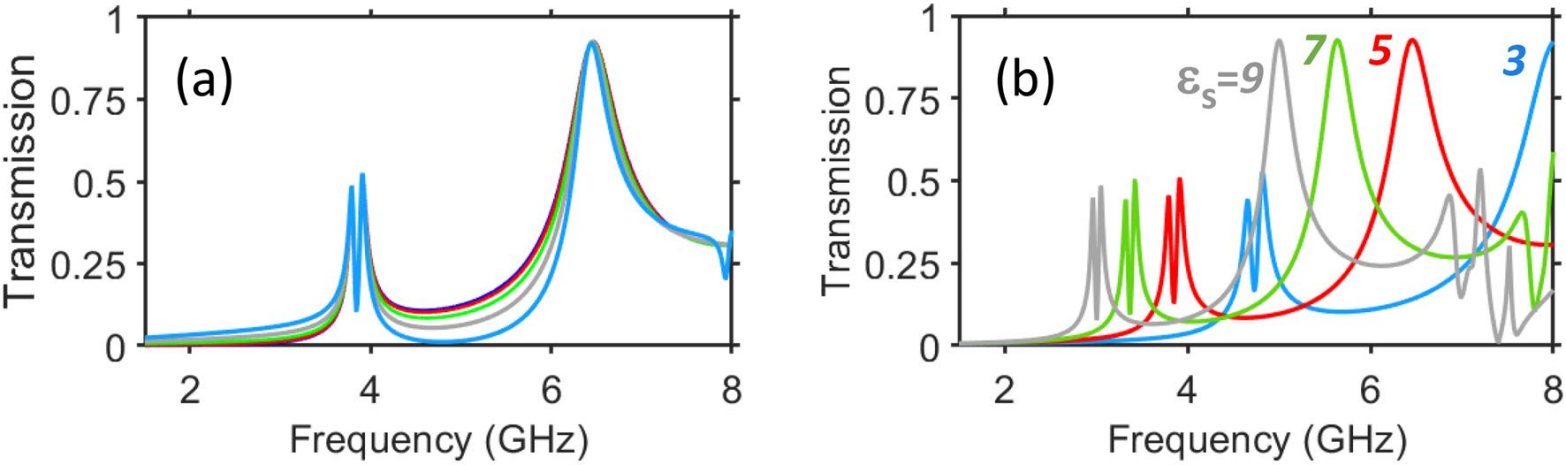
Effects $\theta$ and $\varepsilon _s$ on handedness reversal of CP waves in reflection mode: spectrum of reflection coefficient $\vert {r}_{-+}^{\rightarrow }\vert$ (**a**) at different incidence angles: $\theta =0^{\circ }$ (blue line), $\theta =10^{\circ }$ (red line), $\theta =20^{\circ }$ (light-green line), $\theta =30^{\circ }$ (gray line), $\theta =40^{\circ }$ (light-blue line), when $\varepsilon _s=5$; (**b**) at different values of $\varepsilon _s$: $\varepsilon _s=3$ (light-blue line), $\varepsilon _s=5$ (red line), $\varepsilon _s=7$ (light-green line), $\varepsilon _s=9$ (gray line), when $\theta =0 ^{\circ }$; the remaining parameters are the same as in Figs. 4, 5 and 6. Subscripts + and - stand for left-handed and right-handed CP wave, respectively.

From the multifunctionality perspective, it is worth mentioning that the same structure as in Fig. 4 may enable *reversal* of the handedness of the CP incident waves in reflection mode. As demonstrated in Fig. 9a for $\varepsilon _s=5$, it happens between the first and second transmission-mode PPR bands, which have been observed for LP waves. Interestingly, this effect is robust to the $\theta$-variations. Indeed, $\vert {r}_{-+}^{\rightarrow }\vert$ does not vary, at least when $\theta$ is varied from $\theta =0^{\circ }$ to $\theta =40^{\circ }$. Understanding the mechanism underlying the observed insensitivity is beyond the scope. Notably, various mechanisms of angular insensitivity have been discussed; for instance, see Refs.^31,66,67^. In addition, it is observed in Fig. 9b that the frequency of $\hbox {max}\vert {r}_{-+}^{\rightarrow }\vert$ is approximately scalable by varying $\varepsilon _s$. In particular, the maximum’s frequency is shifted nearly from 8 GHz down to 5 GHz, while $\varepsilon _s$ is varied from 3 to 9, i.e. it is proportional to $1/\sqrt{\varepsilon _s}$. The features observed in Fig. 9a in the vicinity of $f=4$ GHz result from the joint effect of two resonator arrays and the middle grid. In the contrast with it, the ones seen in the vicinity of $f=6.4$ GHz are almost the same as those observed in the case when the back-side resonator array is removed (not shown), so that only the front-side resonators array and the middle grid contribute to the appearance of the maximum of $\vert {r}_{-+}^{\rightarrow }\vert$. Involvement of this regime enhances the overall multifunctionality potential of the studied metasurfaces.

## Conclusion

To summarize, we have studied few-layer metasurfaces comprising two arrays of subwavelength resonators (one of which is rotated by $90^{\circ }$ with respect to the other) and a metallic grid (rectangular-hole array), which jointly enable the efficient conversion of linear polarization to the orthogonal one, in both symmetric and asymmetric transmission regimes. While a geometrically symmetric structure may need the broken time-reversal symmetry or time modulation in order to achieve different forward and backward transmissions, obtaining nearly the same forward and backward cross-polarized transmissions in a structure with broken spatial inversion symmetry is possible rather due to the accurate adjustment of the polarization-conversion-enabling resonances. This opens a route to new multifunctional scenarios that involve both symmetric and asymmetric PPR functionalities at the subwavelength scale. In the suggested nonsymmetric designs, the lower-frequency polarization-conversion band is a conventional AT band, whereas the second-lowest PPR band contains a subrange that can be said to be an “anti-AT”, or a nearly symmetric transmission band. Regarding the symmetric regime, it should be noted that crossing the curves representing *f*-dependencies of the two cross-polarized transmission components is not unique, but it is not trivial to obtain such (nearly) equal but simultaneously high transmittances. This feature is rather unusual for ultrathin metastructures. It is achieved by using the ability of the middle grid to totally change the scenario of arrays coupling compared to the designs without a grid. Moreover, the grid helps to suppress the remaining co-polarized transmission components that are unwanted for AT. As an example, the resulting thickness can be nearly $6.75\times {10}^{-2}\lambda$ ($\lambda$ is free-space wavelength) for the lowest-frequency (first) polarization-conversion band and nearly $0.135\lambda$ for the second band, as occurs for the design in Fig. 4 at $\varepsilon _s=5$. At the same time, the array’s period is about $0.33\lambda$ and $0.66\lambda$, respectively, so that the unwanted diffractions do not appear, at least at zero and small angles of incidence. As follows from the obtained results, the frequencies, at which the desired cross-polarization effects do appear, are scalable by variations of the spacers’ permittivity, and a part of the examined permittivity range can be used for obtaining the targeted combination of the functionalities in one metastructure. Normal incidence is shown to be most suitable, while nonzero angles of incidence can be applicable to other multifunctional scenarios that will be considered in a future paper. It is demonstrated that the nearly symmetric PPR functionality can be achieved within a wide range of the polarization angle variation, whereas the asymmetric PPR functionality and, thereby, its co-existence with the symmetric one can be sensitive to the choice of incident and polarization angles. Design adjustment has been discussed, including the case when materials that are conventional for antennas and other microwave components are used for spacers; the chosen materials and geometrical sizes are consistent with the commonly used fabrication techniques. Moreover, the overall capability of multifunctionality can be enriched by involving the waves with circular polarization. In particular, wide-angle conversion of the wave with left-handed circular polarization to the one with right-handed circular polarization, or vice versa, is achieved in the reflection mode in the same structure, for a wide range of variation of the spacers’ permittivity. A study of the structures comprising the actively tunable components is in progress, as well as an extension of the proposed approach to THz and higher frequencies.

## Methods

The utilized approach is based on the qualitative physical estimates and extensive numerical simulations for the accurately chosen ranges of variation of geometric and material parameters. CST Studio Suite^68^, which is an efficient commercial software program, was used. This numerically efficient program is based on the finite integration method with controllable convergence and accuracy that makes it particularly appropriate for few-layer metasurfaces comprising unit cells of complex geometry. The frequency-domain solver with Floquet–Bloch boundary conditions and a tetrahedral mesh have been used to perform the simulations.

### Supplementary Information

Supplementary Information.

## Data Availability

All of the data generated or analyzed during this study are included in this article and its supplementary information file.
