# Supplementary material for: Few-layer bifunctional metasurfaces enabling asymmetric and symmetric polarization-plane rotation at the subwavelength scale
Source: Sci Rep. 2024 Jun 13;14:13636. doi: 10.1038/s41598-024-62073-4 (PMC11176349; doi:10.1038/s41598-024-62073-4)
Supplement: Supplementary file 1 — Supplementary Information. [file 41598_2024_62073_MOESM1_ESM.pdf]

Supplementary Material

Few-layer bifunctional metasurfaces enabling asymmetric and symmetric polarization-plane rotation at the subwavelength scale

Mutlu Gokkavas, T. F. Gundogdu, Ekmel Ozbay, and Andriy E. Serebryannikov

Although the detailed analysis of the field distribution in the polarization manipulation regimes is beyond scope, we present here several illustrative examples. The differences occurring while changing polarization of the incident wave, i.e., from *y*-polarized to *x*-polarized wave, and vice versa are demonstrated in Figs. S1-S3.

Figure S1 presents the vector maps of the electric and magnetic fields at the lowest-frequency resonance enabling polarization manipulation in the vicinity of *f*=3.8 GHz. For the *y*-polarized incident wave, the features indicating a magnetic dipole behavior of the back-side resonators are observed. However, there are no such pronounced features for the front-side resonators. Accordingly, there is no significant cross-polarized component in the transmitted field. On the contrary, for the *x*-polarized incident wave, we can see resonant effects at the both front- and back-side arrays. Their resonators' coupling is assisted by the effects exerted by the spacer and the mid-grid. These effects may totally change the scenario of coupling of the front-side and the back-side resonator arrays, as compared to the corresponding grid-free case. In this case, a strong cross-polarized component appears. The aforementioned observations are in agreement also with the results in (*x*,*z*)-midplane (not shown). In particular, the results in the incidence/exit half-space in the right lower plot [i.e., in (*z*,*y*)-plane] look similar to those in the exit/incidence half-space in (*x*,*z*)-midplane. In fact, we could recognize signatures of two magnetic dipoles, which can be said to be located above a frequency-selective reflector (which is obtained due to the grid) and coupled owing to its specific properties. As explained by the results in Fig. 3, the difference in transmission efficiency for *x*- and *y*-polarized incident waves is crucial for asymmetric polarization-plane rotation (PPR) functionality and related asymmetric transmission.

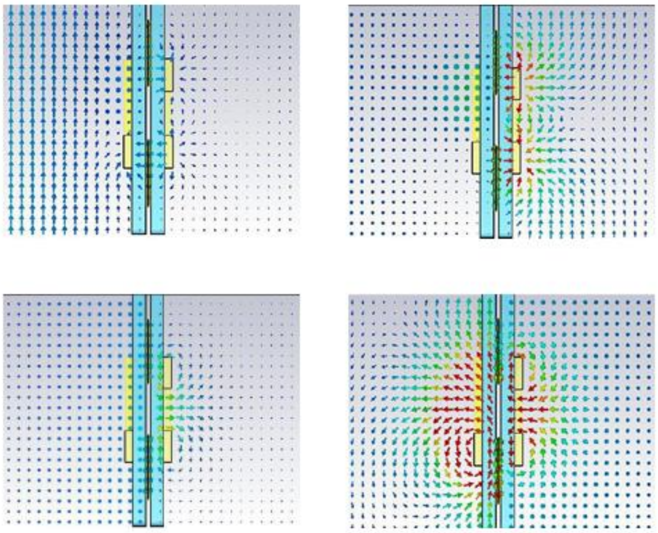

Figure S1. Vector maps for electric (upper plots) and magnetic (lower plots) fields for the basic set of geometric parameters taken from Figs. 1 and 4 and ε_s=5, at the frequency value (*f*=3.8 GHz) corresponding to the first maximum of the cross-polarized component that is observed in the left inset in Fig. 4, for *y*-polarized (left plots) and *x*-polarized (right plots) incident wave; direction of incidence is from the left to the right. Results are presented for the midplane of a front-side resonator, i.e., in (*z*,*y*)-plane (side view).

Next, Fig. S2 presents the vector maps of the electric and magnetic fields at the lower-frequency maximum of the polarization manipulation band, which occurs in the vicinity of *f*=8.9 GHz. Similarly to Fig. S1, Fig. S2 corresponds to the case when polarization conversion is well pronounced for *x*-polarized incident waves but is weak for *y*-polarized waves. The features indicating magnetic-dipole behavior are well seen at the back-side array, in the lower right plot. Although the features observed at the front-side array in this case do not look entirely similar to the ones in Fig. S1, consideration of the (*x*,*z*)-midplane (results are not shown) may lead to the conclusion that the resulting mechanism is similar to the one in Fig. S1, so that the exit-side dipole can be considered as a 90-degrees rotated version of the front-side one.

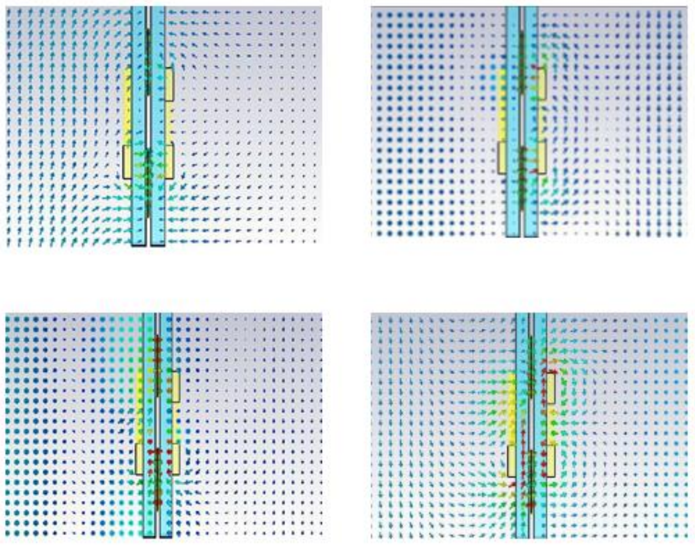

Figure S2. Vector maps for electric (upper plots) and magnetic (lower plots) fields for the basic set of geometric parameters taken from Figs. 1 and 4 and ε_s=5, at the frequency value (*f*=8.9 GHz) corresponding to the first maximum of the cross-polarized component that is shown by blue line in the right inset in Fig. 4, for *y*-polarized (left plots) and *x*-polarized (right plots) electromagnetic wave incident from the left to the right. Results are presented for the midplane of a front-side resonator, i.e., in (*z*,*y*)-plane (side view).

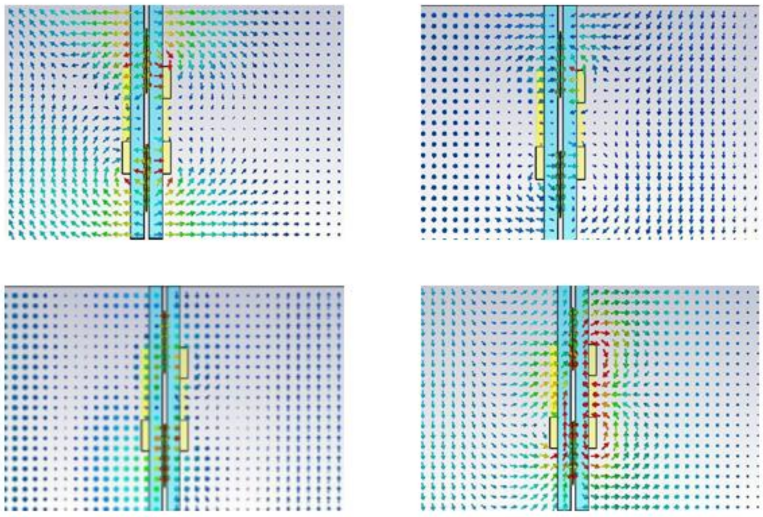

Figure S3. Vector maps for electric (upper plots) and magnetic (lower plots) fields for the basic set of geometric parameters taken from Figs. 1 and 4 and ε_s=5, at the frequency value (*f*=9 GHz) corresponding to the maximum of the cross-polarized component that is shown by red line in the right inset in Fig. 4, for *y*-polarized (left plots) and *x*-polarized (right plots) electromagnetic wave incident from the left to the right. Results are presented for the midplane of a front-side resonator, i.e., in (*z*,*y*)-plane (side view).

Finally, Fig. S3 presents the vector maps of electric and magnetic fields in the vicinity of f=9 GHz, i.e., in the case when the magnitudes of the cross-polarized transmission coefficients for the x-polarized and y-polarized incident waves are close to each other, so that the (nearly) symmetric PPR functionality can be obtained. For the x-polarized waves, the mechanism of polarization conversion is similar to the cases shown in Figs. S1 and S2 for the same polarization. For the y-polarized waves, we observe such a field distribution that has not been observed in other cases considered. It is obvious that the features distinguishing this case from the others include, first of all, strong electric field between the resonators, e.g., at the front-side and back-side surfaces and in the adjusted parts of the incidence and exit half-spaces, respectively, while the results in Fig. 3 may indicate stronger fields in the holes and near the grid. The obtained field maps give us a hint that these features may be connected with the entire-array resonances. Indeed, the fact that the (nearly) symmetric PPR functionality can be obtained within a wide range of variation in ε_s, but needs a careful adjustment of the grid hole size (as shown in Fig. 3) argues in favor of this guess. Note that the rigorous multimode analysis needed to retrieve the detailed pattern of physical processes is beyond scope of this work, but is considered as one of the subjects of our future research.
